# Evaluation of Childhood Atopic Dermatitis and Environmental Factors in Turkey with Decision Tree Model

**DOI:** 10.3390/ijerph22121812

**Published:** 2025-12-02

**Authors:** Nesrullah Ayşin, Mehmet Bulduk, Veysel Can, Eda Nur Muhafiz, Bahattin Bulduk, Emine Kurt Can

**Affiliations:** 1 Vocational School of Health Services, Department of Medical Services and Techniques, Hakkari University, 30000 Hakkari, Turkey; nesrullahaysin@hakkari.edu.tr; 2 Faculty of Health Sciences, Department of Nursing, Van Yüzüncü Yıl University, 65000 Van, Turkey; veyselcan@yyu.edu.tr (V.C.); bahattinbulduk@yyu.edu.tr (B.B.); eminekurtcan@yyu.edu.tr (E.K.C.); 3 Ağrı Taşliçay State Hospital, 04000 Agri, Turkey; edanurmuhafiz@gmail.com

**Keywords:** atopic dermatitis, environmental factors, air pollution, pediatric population, decision tree model

## Abstract

Objective: This study aims to examine the relationship between atopic dermatitis (AD), one of the most common dermatological conditions in children, and environmental factors, including meteorological variables and air pollution. Methods: This retrospective cross-sectional study analyzed the medical records of 21,407 pediatric patients aged 0 to 18 years who presented to the city hospital in Agri, Turkey, between 2020 and 2024. Admission dates were matched with meteorological data (wind speed, atmospheric pressure, humidity, temperature) and air pollution indicators (PM_10_, SO_2_, NO_2_, NOx, NO, O_3_). Statistical analyses included *t*-tests, correlation analyses, binary logistic regression, and a CHAID decision tree model. Results: AD accounted for 10.1% of all dermatology-related visits. AD admissions increased particularly during the first half of the year and were significantly associated with higher O_3_ levels, whereas increased PM_10_ levels were associated with a lower likelihood of AD admissions. Logistic regression showed that age, sex, semiannual period, atmospheric pressure, PM_10_, and O_3_ were significant predictors of AD. The decision tree model identified age, period, and O_3_ as the strongest discriminating variables for AD. Conclusion: AD was found to be more sensitive to environmental and seasonal variations compared with other dermatitis types. In particular, elevated ozone levels and temporal factors played a notable role in increasing AD presentations. These findings may inform environmental risk management and preventive strategies for children with AD.

## 1. Main Message

Atopic dermatitis in children is more sensitive to environmental changes than other dermatitis types, showing a clear increase with rising ozone levels and distinct seasonal variability.

## 2. Background

Air pollution is a complex mixture of certain solid particles, liquid droplets and gas molecules [1]. The U.S. Environmental Protection Agency (EPA) identifies major air pollutants, including ozone (O_3_), particulate matter (PM), carbon monoxide (CO), sulfur dioxide (SO_2_), and nitrogen dioxide (NO_2_), all of which have well-established negative effects on human health [2]. The concentration of these air pollutants has increased significantly in some regions around the world due to sociocultural trends such as increased forest fires, urbanization and industrialization [3]. Today, 91% of the global population lives in areas where air pollution levels exceed the safe exposure limits defined by the World Health Organization (WHO) [4]. Exposure to air pollution can adversely affect human health and increase the risk of AD, a common skin condition known for its sensitivity to environmental triggers, or exacerbate existing symptoms [5,6,7].

AD has become one of the most common inflammatory skin disorders worldwide, and environmental triggers are believed to play a significant role in its development [5,8]. AD, also known as eczema, is a common skin disease that affects 20% of children and 5% of adults globally [9]. This chronic or recurrent inflammatory condition is characterized by sudden flare-ups of eczematous and itchy lesions on dry skin [10,11,12,13]. Recurrent eczema lesions and intense itching can lead to significant impairment of quality of life [14].

Although air pollutants such as NO_2_, SO_2_, NOx, O_3_, and PM_10_ have been reported to be associated with AD prevalence and symptoms, it is noted that these effects may be influenced by confounding factors such as age, gender, geographical location, and economic conditions [15]. Furthermore, it is reported that most of these pollutants are highly correlated with each other [16]. At the cellular level, particulate matter (PM) and polycyclic aromatic hydrocarbons (PAHs) can induce oxidative stress, directly damaging protein and lipid structures in the skin and thereby disrupting the integrity of the skin barrier [17,18]. This oxidative damage leads to dysregulation in both innate and adaptive immune responses, increasing sensitivity to allergens and triggering a cycle in which inflammation and allergic sensitization are continuously sustained [19,20]. Additionally, interactions between environmental pollutants and the neuroimmune system have been shown to play a critical role in itch pathways specific to AD, further highlighting the complex biological relationship between external exposures and cutaneous responses [21,22].

In addition, meteorological conditions (such as humidity, temperature, and wind) also play an important role in increasing the frequency of AD and the severity of its symptoms [23]. Different climatic factors are known to affect the development of postnatal skin functional parameters [24]. Epidemiological studies suggest that common climatic variables are associated with childhood eczema prevalence [25,26,27]. Epidemiological research is important not only for determining the prevalence of diseases but also for planning preventive healthcare services. The exact determination of the frequency of skin diseases is only possible through extensive population studies [28].

This study was conducted to evaluate the most common AD among other types of dermatitis (DDT) and to compare it with DDT. Using patient data collected over a four-year period in eastern Turkey, the effects of air pollution and meteorological variables on these types of dermatitis were investigated in detail. This study, which uses a decision tree model (CHAID) to assess the impact of environmental factors on AD and DDT in a seasonal context, is one of the rare studies of its kind. This unique approach ensures that the findings make a significant contribution to the literature. Moreover, with the four-year dataset, the reliability of the findings is enhanced, and a more comprehensive exploration of the effects of environmental factors on DDT has been made possible. The findings of the study aim to indirectly contribute to the development of individualized care strategies and the improvement of environmental health policies.

## 3. Methods

This study is a retrospective research designed using data obtained from the registry system of a single-center city hospital. The data of children diagnosed with DDT and AD between August 2020 and July 2024 were analyzed in this study.

### 3.1. Study Population and Sample

The study population consisted of children aged 0–18 years who were diagnosed with DDT or AD. No sampling was performed, and data from all patients who presented with these diagnoses to a single-center city hospital between August 2020 and July 2024 were included in the study. This retrospective cross-sectional study analyzed a total of 21,407 patient records from individuals residing in Agri, one of the cities in eastern Turkey with the highest levels of air pollution, who presented to local healthcare facilities. Following the pandemic period, the systematic recording of hospital admissions enabled the construction of a dataset that covered the longest available timeframe including both meteorological variables and air pollution measurements. Within this dataset, atopic dermatitis emerged as the most frequently observed specific dermatitis subtype. Examination of all dermatitis diagnoses showed that many subtypes occurred at extremely low frequencies and did not constitute epidemiologically meaningful groups. In contrast, atopic dermatitis with 2151 cases corresponding to 10.1 percent was the only specific dermatitis diagnosis with a substantial prevalence. The remaining dermatitis categories were either broad and heterogeneous or had case numbers below one percent, making them unsuitable for separate analytical grouping. For this reason, patients were classified into two main diagnostic categories. The DDT group consisted of 19,256 cases representing 89.9 percent while the AD group included 2151 cases representing 10.1 percent of all dermatitis related visits. This classification approach was chosen to strengthen statistical power and to allow a more accurate evaluation of atopic dermatitis as the only subtype with a meaningful epidemiological burden.

The first category consisted of demographic variables. Patient information was obtained from electronic health records after the necessary permissions were granted by the Agri Provincial Health Directorate. The second category included meteorological parameters such as wind, pressure, humidity, and temperature, and these data were obtained from the Agri Provincial Directorate of Meteorology. The third category comprised air pollution variables including NO_2_, SO_2_, NOx, O_3_, and PM_10_, which were also provided by the Agri Provincial Directorate of Meteorology. All meteorological and air pollution variables were included in the analyses as continuous variables, and their monthly mean values were used. Demographic, meteorological, and air pollution data were obtained directly from the relevant institutions, and the study period was defined as August 2020 to July 2024. This timeframe was selected because it represented the most consistent and uninterrupted period during which both hospital records and meteorological and air pollution measurements were systematically available.

### 3.2. Inclusion Criteria

- Children and adolescents aged zero to eighteen years
- Individuals residing in the central district of Agri province
- Patients who presented to the Agri city hospital with dermatological complaints
- Records with complete and accurate information including age, sex, diagnosis code and date of admission
- Patients diagnosed with dermatitis belonging to the DDT or AD categories, selected because these were the only clinically meaningful and sufficiently frequent diagnostic groups in the dataset

### 3.3. Exclusion Criteria

- Patients who did not reside in the central district of Agri
- Records with missing, incomplete or incorrect information
- Visits made for reasons other than dermatological conditions
- Dermatitis diagnoses outside the DDT and AD categories, since these remaining ICD codes had very low frequencies or represented heterogeneous conditions unsuitable for analytical grouping

### 3.4. Data Collection Method

Patient data were retrospectively obtained from the electronic registry system of the city hospital. The data included information such as the patient’s age, gender, application date, and diagnosis type (DDT or AD). Meteorological data (temperature, humidity, pressure, wind speed) and air quality measurements (concentrations of PM_10_, NO_2_, SO_2_, etc.) were provided by national meteorological and environmental air quality monitoring systems. The meteorological and air quality data were matched with patient application dates, and the analyses were performed using monthly averages.

### 3.5. Data Analysis

All statistical analyses in this study were performed using the SPSS 26.0 software package, and the analytical process was designed with a multidimensional evaluation approach. In the first stage, the descriptive characteristics of the sample were summarized through frequencies, percentages, means, and standard deviations, and Pearson Chi Square tests were used to examine differences between categorical variables. Relationships between the independent variables and the binary dependent variable were examined using the Spearman correlation coefficient, and Cramer V effect size was calculated to support the interpretation of statistical significance given the large sample size. Independent sample *t*-tests were applied after confirming that meteorological and air pollution variables met the assumption of normal distribution.

In the second stage, a binary logistic regression model was constructed to identify factors predicting atopic dermatitis. All independent variables were entered simultaneously into the model using the enter method, and humidity and temperature variables were excluded due to high multicollinearity. The period variable was included as the only temporal predictor because it showed strong associations with season and month variables. Model assumptions were verified using the Box Tidwell test and VIF values, and potential outliers were assessed with Cook distance diagnostics. Model performance was evaluated using the Omnibus test, minus two Log Likelihood, Cox Snell and Nagelkerke R square values, and calibration was assessed with the Hosmer Lemeshow test. In the third stage, the CHAID decision tree model was applied to explore interactions between variables and to reveal hierarchical segmentation. Model generalizability was ensured through the use of Bonferroni corrected Chi Square splitting criteria and tenfold cross validation. All analyses were conducted with a ninety five percent confidence level, and statistical significance was accepted as *p* less than 0.05.

### 3.6. Ethical Approval

Ethical approval for the study was obtained from the Ethical Committee for Non-Interventional Clinical Research of Van Yzüncü Yıl University on 18 September 2023 (decision number 2023/09-18). The data used in the study were anonymized, and the confidentiality of personal information was maintained.

## 4. Results

Of the 21,407 pediatric hospital records analyzed, 19,256 (90%) were diagnosed with dermatitis and 2151 (10%) with AD. Among girls, 91% were diagnosed with dermatitis and 8.6% with AD, whereas the corresponding rates for boys were 85.5% and 11.5%, respectively. Girls accounted for 50.8% of all dermatitis cases, while boys constituted 57.4% of all AD cases. A statistically significant yet small effect was observed between sex and diagnosis type (χ^2^ = 52.161, *p* < 0.001, V = 0.02), with a weak positive correlation (r = 0.049, *p* < 0.001).

The age of children with dermatitis ranged from 1 to 19 years (mean = 6.4 ± 5.6), while the age range for those with AD was 1 to 18 years (mean = 3.8 ± 4.5). Children with AD were significantly younger (t = 20.641, *p* < 0.001, η^2^ = 0.03). A weak and negative correlation was detected between age and diagnosis type (r = −0.159, *p* < 0.001), indicating that younger children were more likely to receive an AD diagnosis.

Across the four annual periods examined (August 2020–July 2024), total hospital visits increased steadily, with the August 2023–July 2024 period (*n* = 7552) showing nearly twice as many visits as the first period (*n* = 3763). Dermatitis consultations rose from 17.7% in the first period to 33.8% in the fourth. Nearly half of all AD visits (48.6%) occurred in the final period. Period and diagnosis type were significantly associated (χ^2^ = 211.284, *p* < 0.001, V = 0.03), and their correlation was weak and positive (r = 0.063, *p* < 0.001).

In the semiannual analysis, dermatitis rates were similar between the first and second six-month periods (49.7% vs. 50.3%). In contrast, AD was more common during the first six months (58.1%). The difference was statistically significant with a small effect size (χ^2^ = 211.284, *p* < 0.001, V = 0.02). A weak negative correlation was observed between semiannual period and diagnosis type (r = −0.051, *p* < 0.001).

Seasonal distribution of dermatitis was relatively stable (winter 25.9%, spring 24.6%, summer 25.3%, autumn 24.2%). Atopic dermatitis rates were highest in spring (29.7%) and lowest in autumn (19.3%). The association between season and diagnosis type was statistically significant with a small effect size (χ^2^ = 39.476, *p* < 0.001, V = 0.01), accompanied by a weak negative correlation (r = −0.027, *p* < 0.001).

In the monthly analysis, February and May showed higher proportions of AD, whereas August, September, November, and December exhibited higher proportions of dermatitis. Month and diagnosis type were significantly associated (χ^2^ = 75.638, *p* < 0.001), with a weak negative correlation (r = −0.043, *p* < 0.001). (Table 1).

**Figure 1:**
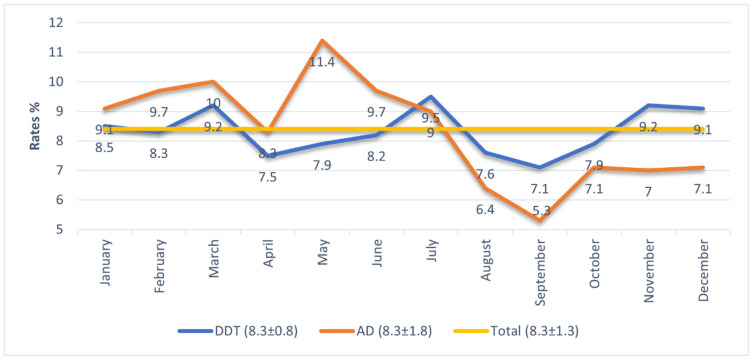
Proportional registration of hospital admissions diagnosed with DDT and AD by months.

As shown in Figure 1, the mean monthly column percentages for dermatitis and AD were both 8.3% (dermatitis SD = 0.8; AD SD = 1.8; total SD = 1.3), indicating similar month-to-month distribution patterns.

It is observed that hospital admission rates for atopic diagnoses generally remain above both dermatitis diagnoses and overall mean admission rates; however, in July, the admission rate for atopic cases falls below that of dermatitis, and in August, September, November, and December, it declines further, remaining below both dermatitis and overall averages. Examination of the standard deviation values shows that while monthly dermatitis admissions have a standard deviation of 0.8, atopic admissions have a higher standard deviation of 1.8, indicating that atopic admissions fluctuate more across months. In the first six months (January–June), atopic admission rates are higher than dermatitis admissions, peaking in May (11.4%). The month of July represents a turning point where atopic admission rates begin to decline, reaching their lowest point in September (5.3%). (Figure 1).

Wind Speed

A significant difference was observed between the groups in terms of mean wind speed (t = –2.279, *p* = 0.023). The average wind speed during periods with AD visits was higher (M = 14.6) compared to periods with dermatitis visits (M = 14.3). Wind speed showed a positive correlation with AD visit frequency (r = 0.028, *p* < 0.001).

In the first period (January–June), dermatitis visits were associated with higher wind speeds (M = 15.4 vs. M = 15.2; t = 2.155, *p* = 0.031). In the second period (July–December), wind speed was higher during AD visits (M = 13.7 vs. M = 13.4; t = –2.904, *p* = 0.004). Seasonally, wind speed was significantly higher during dermatitis visits in spring (t = 2.155, *p* < 0.05) and during AD visits in winter (t = –2.333, *p* < 0.05), while no differences were observed in summer or autumn (*p* > 0.05). Monthly analyses indicated higher wind speeds for dermatitis in March (t = 2.559), April (t = 2.991), and June (t = 2.274), whereas higher wind speeds for AD visits were found in September (t = –2.224), November (t = –4.099), and December (t = –3.266) (all *p* < 0.05).

Atmospheric Pressure

Mean atmospheric pressure differed significantly between groups (t = 3.835, *p* < 0.001). The pressure on days with AD visits was lower (835.21) compared to days with dermatitis visits (835.41), and higher pressure was associated with fewer AD visits (r = –0.023, *p* < 0.001). In the first period, mean pressure was significantly higher during dermatitis visits (M = 836.3 vs. M = 835.1; t = 2.509, *p* = 0.011). Seasonal analysis showed a significant difference only in winter (t = 4.879, *p* < 0.05). Monthly comparisons revealed higher pressure during dermatitis visits in January (t = 3.313), February (t = 2.333), and March (t = 3.943) (all *p* < 0.05).

Humidity

There was no overall significant difference in mean humidity between the groups (t = –1.823, *p* = 0.085), and no correlation was detected (r = 0.006, *p* > 0.05). However, in the first period, humidity was significantly higher during dermatitis visits (M = 66.5 vs. M = 65.6; t = 2.727, *p* = 0.006). Seasonal analysis indicated higher humidity for dermatitis in spring (t = 4.275, *p* < 0.05) and for AD visits in autumn (t = –2.787, *p* < 0.05).

Monthly analyses showed higher humidity for dermatitis in April (t = 6.678), while higher humidity for AD visits was observed in September (t = –2.645) and October (t = –4.069) (all *p* < 0.05).

Temperature

No overall difference was found in mean temperature between the groups (t = 0.080, *p* = 0.936), and no correlation emerged (r = –0.001, *p* > 0.05). In the first period, temperature was significantly higher during AD visits (M = 5.6 vs. M = 4.9; t = –2.493, *p* = 0.001). Seasonally, a significant difference was observed only in spring, with higher temperatures during AD visits (t = –4.275, *p* < 0.05). Monthly analyses revealed higher temperatures for AD visits in March (t = –3.041), April (t = –6.594), May (t = –1.986), and June (t = –4.031), while higher temperatures for dermatitis visits were detected in September (t = 2.848) and October (t = 3.859) (all *p* < 0.05). (Table 2).

PM_10_

Mean PM_10_ levels differed significantly between the groups (t = 4.564, *p* < 0.001). PM_10_ was lower on days with AD visits (56.9 < 60.6), and higher PM_10_ levels were associated with fewer AD visits (r = –0.023, *p* < 0.001). In Period 1 (January–June), PM_10_ was higher during dermatitis visits (M = 59.2 vs. 55.9; t = 2.806, *p* = 0.005). In Period 2 (July–December), PM_10_ remained higher for dermatitis (M = 62.0 vs. 58.3; t = 2.693, *p* = 0.007). Seasonally, significant differences appeared in autumn (t = 4.472) and winter (t = 3.829), with higher PM_10_ during dermatitis visits (*p* < 0.05). Monthly analyses showed higher PM_10_ for dermatitis in January, February, October, and November; and higher PM_10_ for AD visits in April, June, July, and August (*p* < 0.05).

SO_2_

A significant difference was found between the groups (t = 2.864, *p* = 0.004), with lower SO_2_ levels on AD visit days (13.0 < 13.8). SO_2_ increases were associated with fewer AD visits (r = –0.017, *p* < 0.05). In Period 1, SO_2_ was higher during dermatitis visits (M = 15.5 vs. 14.1; t = 3.849, *p* < 0.001). Seasonal differences were significant in spring (t = 5.895) and summer (t = 3.294), with higher SO_2_ during dermatitis visits (*p* < 0.05). Monthly analyses showed significantly higher SO_2_ for dermatitis in January, March, May, August, and October (*p* < 0.05).

NO_2_

Mean NO_2_ levels differed significantly (t = 3.760, *p* < 0.001), with lower NO_2_ on AD visit days (11.1 < 11.5). Higher NO_2_ was associated with fewer AD visits (r = –0.024, *p* < 0.05). In Period 1, NO_2_ was higher during dermatitis visits (M = 11.4 vs. 10.9; t = 4.264, *p* < 0.001). Significant seasonal differences in spring (t = 6.131) and summer (t = 2.218) indicated higher NO_2_ for dermatitis (*p* < 0.05). Monthly analyses showed significantly higher NO_2_ for dermatitis in March and June (*p* < 0.05).

NO_x_

Groups differed significantly in NOx levels (t = 4.707, *p* < 0.001). Atopic visits occurred on days with lower NOx (16.4 < 17.2), and NOx was negatively correlated with AD visits (r = –0.033, *p* < 0.001). In Period 1, NOx was higher during dermatitis visits (M = 16.1 vs. 15.3; t = 4.988, *p* < 0.001). Seasonally, NOx differences were significant in spring (t = 6.270) and summer (t = 2.050), favoring dermatitis (*p* < 0.05). Monthly comparisons showed higher NOx for dermatitis in March, April, June, and August; and higher NOx for AD visits in July, November, and December (*p* < 0.05).

NO

A significant difference was observed (t = 5.301, *p* < 0.001), with lower NO levels on AD visit days (5.2 < 5.6). Higher NO was associated with fewer AD visits (r = –0.030, *p* < 0.001). In Period 1, NO was higher during dermatitis visits (M = 4.7 vs. 4.4; t = 4.988, *p* < 0.001). Seasonal differences were significant in spring (t = 6.434) and winter (t = 2.750), showing higher NO during dermatitis visits (*p* < 0.05). Monthly analyses revealed higher NO for dermatitis in January, March, April, June, and September; and higher NO for AD visits only in December (*p* < 0.05).

O_3_

O_3_ differed significantly between groups (t = –6.600, *p* < 0.001). O_3_ levels were higher on days with AD visits (74.9 > 70.4), and O_3_ showed a positive correlation with AD visit frequency (r = 0.051, *p* < 0.001). In Period 1, O_3_ was higher during AD visits (M = 77.1 vs. 72.8; t = –7.459, *p* < 0.001). In Period 2, O_3_ remained higher for AD visits (M = 71.9 vs. 68.1; t = –2.870, *p* = 0.004). Seasonally, O_3_ was significantly higher for AD visits in spring (t = –9.800), summer (t = –2.726), and winter (t = –3.421) (*p* < 0.05). Monthly analyses indicated higher O_3_ for AD visits in January, March, April, May, June, July, and November (*p* < 0.05). (Table 3).

The binary logistic regression model was statistically significant (χ^2^(11) = 660.230, *p* < 0.001), explaining 6.3% of the variance (Nagelkerke R^2^ = 0.063; Cox and Snell R^2^ = 0.030) with a −2 Log Likelihood of 13,304.724. The Hosmer–Lemeshow test was significant (*p* < 0.05), indicating limited model fit, likely due to the very large sample size (*n* = 21,407) and class imbalance, as dermatitis cases were classified correctly at 100% while AD cases were classified at 0%. Six of the eleven predictors were significant. Girls had lower odds of receiving an AD diagnosis compared with boys (B = −0.249, Wald = 28.669, *p* < 0.001, OR = 0.779, 95% CI [0.711, 0.854]), whereas younger age increased AD risk (B = −0.104, Wald = 366.494, *p* < 0.001, OR = 0.901, 95% CI [0.891, 0.911]). Visits occurring in July–December were associated with lower AD likelihood (B = −0.466, Wald = 69.793, *p* < 0.001, OR = 0.601, 95% CI [0.533, 0.677]). Among meteorological variables, pressure increased AD odds (B = 0.113, Wald = 29.651, *p* < 0.001, OR = 1.119, 95% CI [1.075, 1.166]), while air pollution measures showed that PM_10_ reduced (B = −0.004, Wald = 16.919, *p* < 0.001, OR = 0.996, 95% CI [0.994, 0.998]) and O_3_ increased AD risk (B = 0.015, Wald = 68.705, *p* < 0.001, OR = 1.015, 95% CI [1.011, 1.018]). Wind speed, SO_2_, NO_2_, NOx, and NO were not significant predictors (*p* > 0.05). (Table 4).

Regression Tree

In the logistic regression analysis conducted based on the number of dermatitis and AD disease cases, the statistically significant variables of gender, period, age, pressure, PM_10_, and O_3_ were included in the classification tree model. The maximum tree depth was set to 3 to facilitate reading/understanding, while the Parent Node and Child Node were kept at their default values. Ten-fold cross-validation was applied to validate the model. The model’s risk prediction is 10.0%. This rate is the same for both resubstitution and cross-validation. This indicates that the model is reasonable for evaluation purposes. The model classified the dependent variable Dermatitis with 100% accuracy according to the Atopic type. However, it failed to classify the “Atopic” category correctly, achieving 0% success. The overall accuracy rate is 90.0%. This result indicates that the model was affected by the imbalanced dataset (the number of Dermatitis cases is very large). Consequently, the model followed a path that predicted the majority class (Dermatitis) instead of the minority class (Atopic). Ultimately, the classification tree produced a view consisting of age, period, and O_3_ data (Table 5).

**Figure 2:**
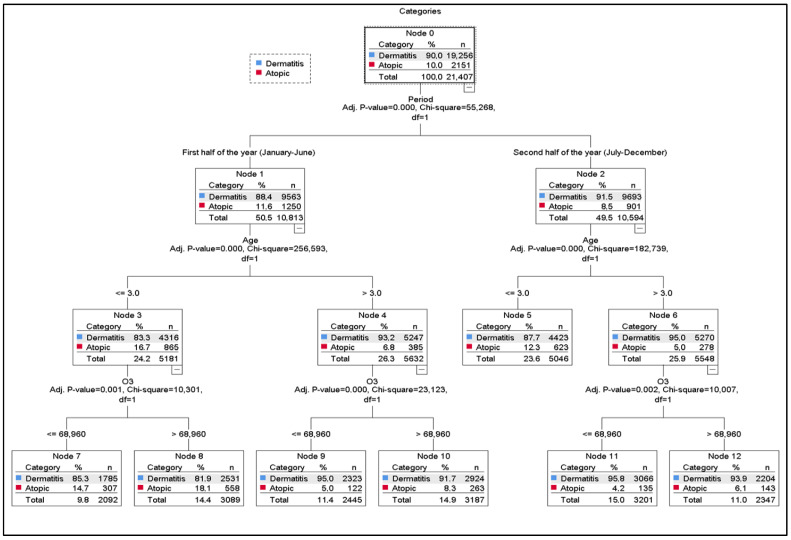
Classification Tree. Performance nodes and accuracy rates. For DDT. Node 11 (95.8 percent), Node 9 (95.0 percent), Node 12 (93.9 percent), Node 10 (92.0 percent), Node 5 (97.4 percent), Node 7 (94.9 percent), Node 8 (91.1 percent). For AD. Node 8 (18.1 percent), Node 7 (14.7 percent), Node 5 (12.3 percent), Node 10 (12.2 percent), Node 12 (6.6 percent), Node 9 (5.0 percent), Node 11 (4.2 percent).

Figure 2 shows that the root node (Node 0) contains 90.0% dermatitis and 10.0% AD cases. The first split is based on the period variable, which is statistically significant (χ^2^(1) = 55.3, *p* < 0.001). AD prevalence is higher in the first half of the year (11.6%) and decreases in the second half (8.5%).

Both periods are subsequently divided by age (χ^2^(1) = 255.6 and χ^2^(1) = 182.7; *p* < 0.001). Children aged ≤ 3 years display the highest AD proportions (Node 3 = 16.7%, Node 5 = 12.3%). Regardless of the period, AD is consistently more common in younger children (≤3 years) than in older ones.

In the first half of the year, both age groups are further split by O_3_ levels (χ^2^(1) = 10.3 and χ^2^(1) = 23.1; *p* < 0.05), with an O_3_ threshold of 68.96. In all nodes, O_3_ values above this threshold correspond to higher AD proportions, whereas lower levels correspond to higher dermatitis proportions.

Among children ≤ 3 years in the first period, AD rates are 14.7% when O_3_ is below the threshold (Node 7) and 18.1% when above it (Node 8). Among children >3 years, AD is 5.0% below the threshold (Node 9) and 8.3% above it (Node 10). In the second period, AD rates for children >3 years rise from 4.2% (Node 11) to 6.1% (Node 12) when O_3_ exceeds the threshold.

The nodes with the highest AD representation are Node 8 (18.1%) and Node 7 (14.7), both corresponding to children ≤ 3 years in the first half of the year. Although O_3_ distinguishes these nodes, the strong influence of age suggests that age remains the dominant predictor over O_3_ (Figure 2).

## 5. Discussion

This study, while examining the relationship between AD and environmental factors such as air pollution and meteorological parameters, also evaluated other DDT types in a general context. The findings suggest that AD shows a more distinct sensitivity to environmental factors, and overall trends related to DDT types were also presented. Of the total admissions included in the study, 90% were children diagnosed with DDT, and 10% were children diagnosed with AD.

A negative correlation was found between age and AD diagnoses, with a tendency for the number of AD diagnoses to increase as age decreases. Literature reports that AD first appears as early as 1 month of age, with its prevalence increasing with age and peaking at 2.5 years old [29,30]. These findings suggest that AD may be more commonly observed in early childhood.

The study found that AD diagnosis was more common in males, and this difference was statistically significant. Research in the literature also supports that the prevalence of AD in early childhood tends to be higher in males [31,32]. However, as children grow older, this trend changes, and increased sensitivity is observed among females during adolescence and adulthood [33]. In the 0–2 age group, AD was more frequently seen in males, but in the 12–16 age group, this trend reversed, with higher prevalence in females [28].

In the study, AD admissions in 2023–2024 showed a slight increase during the first three years, but in the fourth year, they almost tripled, accounting for 48.6% of the total AD admissions. Recent studies have shown a marked increase in AD consultations, particularly among pediatric populations, which can be attributed to increased awareness and improved diagnostic practices [32,34]. In recent years, there has been a reported increase in the prevalence of AD in children, with studies indicating that environmental factors, such as urban living and exposure to allergens, play a significant role in this rise [9,32,35].

The seasonal distribution of AD is characterized by fluctuations in symptom severity influenced by environmental factors such as temperature, humidity, and allergen exposure [36]. The study found that there were significantly more AD admissions in the first six months compared to the second half of the year. Seasonal flare-ups of AD are commonly observed, and low ambient humidity and cold winter air exacerbate the severity of symptoms [37]. Low humidity levels and cold air increase transepidermal water loss, leading to skin dryness, and this has been associated with increased symptoms during winter in cold climates [38]. Exposure to allergens during the spring and summer months can trigger AD flare-ups, with symptoms reaching their peak, especially during these seasons [13].

In the study, it was found that AD admissions were higher in February and May compared to other periods. Similarly, studies have reported more frequent occurrences of AD during the spring [39]. However, different findings also indicate that AD is more common in the winter months [28]. These differences suggest that seasonal effects may vary depending on regional and environmental factors, and pollutants such as particulate matter (PM), formaldehyde, and volatile organic compounds can impair the function of the epidermal barrier and intensify AD symptoms [40,41]. The study found that during periods of high wind speeds, AD admissions increased, and a significant positive relationship between wind speed and AD admissions was identified. The literature indicates that wind speed can increase outpatient applications for AD [42]. However, one study reported a negative relationship between wind speed and AD symptoms [43]. Another study found no relationship between wind speed and AD [44]. These differing results suggest that the effect of wind speed on AD should be evaluated alongside other environmental factors, as symptoms may be shaped by the interaction of various environmental conditions.

In the literature, there are studies indicating that high humidity can alleviate AD symptoms [39,45]. However, there are also studies suggesting that high humidity could be an aggravating factor for symptoms [27,46]. Moreover, it was reported that low humidity increases the risk of outpatient visits for childhood AD [42]. In this study, no relationship was found between humidity and AD. These findings suggest that humidity alone is not effective, and its role in symptoms should be evaluated in conjunction with other environmental conditions.

There was no clear relationship between atmospheric pressure and healthcare utilization measurements [36]. However, in this study, it was observed that a unit increase in pressure increased AD visits by 1.117 times. Similarly, another study reported that an increase in atmospheric pressure was associated with an increase in AD applications [47]. These data suggest that further research is needed to understand the effects of atmospheric factors on AD.

Air pollutants, especially PM_10_, SO_2_, and NO_2_, were associated with AD symptoms both in the short and long term. Pollutants like NO_2_ increase AD prevalence with long-term exposure, while PM_10_ and SO_2_ lead to more severe symptoms with short-term exposure [7]. A systematic review on PM_10_ found a significant relationship between exposure to air pollution and increased medical visits due to AD [4]. Findings related to PM_10_ are consistent with previous studies. In one study, every 10 μg/m^3^ increase in PM_10_ levels was associated with a 1.08% increase in AD outpatient visits [48]. Similarly, another study found that every 10 μg/m^3^ increase in PM_10_ levels led to a 4.72% increase in AD visits [49]. In this study, PM_10_ shows a positive correlation with DDT visits. These findings suggest that PM_10_ exposure may be related to DDT symptoms, and the effects of air pollution on symptoms should be evaluated in more comprehensive research.

The study found a negative correlation between SO_2_ levels and AD applications. SO_2_ exposure has been shown to be associated with an increase in AD hospital visits and worsening symptoms [50,51]. The study found a negative correlation between SO_2_ levels and AD applications. However, the literature indicates that SO_2_ exposure is linked to increased hospital visits and worsening symptoms in AD patients [50,51]. These different findings suggest that the effect of SO_2_ on AD may vary depending on environmental conditions, exposure duration, and individual differences. Future research could provide more detailed insights into the complex relationship between SO_2_ and AD.

A negative correlation between NO_2_ levels and AD visits was observed in this study. Two studies conducted in China found an increase in outpatient visits for AD in children during periods of high NO_2_ levels [23,52]. However, a study in Korea found no relationship between NO_2_ and AD [50]. These contradictory findings suggest that the effects of NO_2_ on AD may vary based on regional environmental conditions, the composition of air pollution, and individual sensitivities.

In this study, a negative correlation was found between NOx levels and AD applications. Other studies have reported that NOx is associated with lifetime and past-year prevalence of AD and its effect on eczema incidence [53,54]. This suggests that the impact of NOx on AD may be shaped by different environmental conditions and exposure characteristics. Future studies are needed to better understand the effects of NOx.

A positive correlation between O_3_ levels and AD applications was found in this study. Literature suggests that acute exposure to O_3_ is associated with an increase in daily hospital visits due to AD and that air pollutants can be a major trigger for AD flare-ups [51]. However, some studies have found no relationship between O_3_ and daily hospital visits for AD [50]. This suggests that the effects of O_3_ on AD may depend on factors such as study methods, exposure levels, and environmental conditions.

The decision tree model used in the study evaluated the effects of air pollution and meteorological parameters on AD based on the two halves of the year. The model showed that in the first half of the year (January–June), an increase in O_3_ levels was associated with a significant increase in the number of AD patients. Moreover, when O_3_ levels were below 74,160, an increase in atmospheric pressure led to a significant rise in AD patient numbers. This supports the triggering effects of O_3_ exposure [51] and pressure [47] on AD.

In the second half of the year (July–December), it was observed that PM_10_ levels had a significant effect on the number of AD patients. The model revealed that when PM_10_ levels were above 55,730, an increase in wind speed resulted in an 80% increase in AD patients. However, a decrease in the number of DDT diagnoses was observed during the same period. These findings suggest that wind spreads [42] PM_10_ particles [4] in the atmosphere, increasing AD cases, but this effect is different in DDT types.

Furthermore, the model demonstrated that in the first half of the year, O_3_ and pressure levels, and in the second half, PM_10_ and wind speed, could predict AD patient numbers by 90%. This highlights that air pollution and meteorological factors are important parameters to consider in the management of AD. The decision tree model used in this study stands out with its 90% predictability rate compared to similar studies in the literature. The model provided the opportunity to assess the effects of air pollution and meteorological parameters seasonally and showed that these factors are crucial in patient management. Reducing environmental pollution and managing meteorological variables could be a critical strategy in controlling AD symptoms. In this process, nurses and other healthcare professionals can contribute to patient management by raising awareness and conducting educational programs to reduce environmental exposure. Developing individualized care plans could be an effective approach to minimizing the impact of such environmental and meteorological variables on health.

## 6. Conclusions

The decision tree model (CHAID) used in this study evaluated the effects of air pollution and meteorological factors on AD and DDT types based on seasonal differences and highlighted that these parameters are important factors to consider in disease management. The findings showed that in the first half of the year, O_3_ and pressure levels, and in the second half, PM_10_ and wind speed significantly influenced AD applications. Furthermore, the model’s 90% predictability rate provides a practical tool to evaluate the effects of air pollution and meteorological variables and to develop preventive strategies.

Reducing environmental pollution and managing meteorological variables is crucial in controlling AD symptoms. In this context, nurses and other healthcare professionals can play an effective role in educational programs aimed at reducing environmental exposure through individualized care plans and awareness campaigns. Future studies should comprehensively investigate the effects of air pollution and meteorological factors on DDT by conducting long-term data analysis in different regions.

## 7. Study Limitations

This study has several important limitations that should be acknowledged to correctly interpret the findings. First, the retrospective design does not allow for establishing causal relationships between air pollution, meteorological variables, and AD outcomes; only associations could be evaluated. Second, although monthly averages of meteorological and air quality indicators were used, this approach may mask short-term peaks or fluctuations, such as daily or hourly changes, which could influence symptom exacerbations. Third, the study was conducted in a single-center provincial hospital, which may limit the generalizability of the results to other regions with different climatic, environmental, or demographic characteristics.

In addition, several unmeasured confounding factors may have influenced the outcomes but could not be controlled for due to data limitations, including aeroallergen concentrations (pollen, mold), indoor environmental exposures, socioeconomic status, parental smoking, heating methods, and individual treatment or medication use. Future multicenter studies using prospective designs, enriched datasets, and high-resolution environmental measurements are needed to validate and expand these findings.

## Figures and Tables

**Table 1:** Statistics on DDT and AD data and comparisons.

	DDT			AD			Total		Statistics
	*n*	% Column	% Row	*n*	% Column	%	*n*	% Column	% Row
Gender									
Girl	9790 ^a^	50.8	91.1	917 ^b^	42.6	8.6	10,707	50.0	χ^2^ = 52.161
Boy	9466 ^a^	49.2	88.5	1234 ^b^	57.4	11.5	10,700	50.0	*p* = 0.000 * r = 0.049 ** V = 0.02
Hospital Application Period									
Aug. 2020–Jul. 2021	3405 ^a^	17.7	90.5	358 ^a^	16.6	9.5	3763	17.6	χ^2^ = 211.284 *p* = 0.000 * r = 0.063 ** V = 0.03
Aug. 2021–Jul. 2022	3846 ^a^	20.0	91.4	361 ^b^	16.8	8.6	4207	19.7	
Aug. 2022–Jul. 2023	5499 ^a^	28.6	93.4	386 ^b^	17.9	6.6	5885	27.5	
Aug. 2023–Jul. 2024	6506 ^a^	33.8	86.1	1046 ^b^	48.6	13.9	7552	35.3	
6-Month Period of Hospital Applications									
First Six Months (January–June)	9563 ^a^	49.7	88.4	1250 ^b^	58.1	11.6	10,813	51	χ^2^ = 55.256 *p* = 0.000 * r = −0.051 ** V = 0.02
Second Six Months (July–December)	9693 ^a^	50.3	91.5	901 ^b^	41.9	8.5	10,594	49	
Season of Hospital Application									
Winter	4993 ^a^	25.9	90.0	556 ^a^	25.8	10.0	5549	25.9	χ^2^ = 39.476 *p* = 0.000 * r = −0.027 ** V = 0.01
Spring	4733 ^a^	24.6	88.1	639 ^b^	29.7	11.7	5372	25.1	
Summer	4873 ^a^	25.3	90.0	540 ^a^	25.1	10.0	5413	25.3	
Autumn	4657 ^b^	24.2	91.8	416 ^b^	19.3	8.2	5073	23.7	
Month of Hospital Application									
January	1645 ^a^	8.5	89.4	195 ^a^	9.1	10.6	1840	8.6	χ^2^ = 75.638 *p* = 0.000 * r = −0.043 ** V = 0.02
February	1604 ^a^	8.3	88.5	208 ^b^	9.7	11.5	1812	8.5	
March	1771 ^a^	9.2	89.2	215 ^a^	10.0	10.8	1986	9.3	
April	1438 ^a^	7.5	89.0	178 ^a^	8.3	11.0	1616	7.5	
May	1524 ^a^	7.9	86.1	246 ^b^	11.4	13.9	1770	8.3	
June	1581 ^a^	8.2	88.4	208 ^a^	9.7	11.6	1789	8.4	
July	1828 ^a^	9.5	90.4	194 ^a^	9.0	9.6	2022	9.4	
August	1464 ^a^	7.6	91.4	138 ^b^	6.4	8.6	1602	7.5	
September	1370 ^a^	7.1	92.4	113 ^b^	5.3	7.6	1483	6.9	
October	1512 ^a^	7.9	90.9	152 ^a^	7.1	9.1	1664	7.8	
November	1775 ^a^	9.2	92.2	151 ^b^	7.0	7.8	1926	9.0	
December	1744 ^a^	9.1	91.9	153 ^b^	7.1	8.1	1897	8.9	
Total	19,256	89.9	90.0	2151	10.1	10.0	214,407	100.0	
	**Mean ± ss**	**Min.–Max**		**Mean ± ss**	**Min.–Max**		**Mean ± ss**	**Min.–Max**	
Age	6.4 ± 5.6	1–19		3.8 ± 4.5	1–18		6.1 ± 5.6	1–19	t = 20.641 *p* = 0.000 * r = −0.159 ** η^2^ = 0.03

χ^2^ = Chi-square test; ^a,b^ = Pairwise group differences; V = Cramer’s V (effect size); t = Independent samples *t*-test (normality criterion: skewness and kurtosis within ±2.00); r = Spearman’s rho correlation coefficient; Min = Minimum; Max = Maximum; ** *p* < 0.001; * *p* < 0.05

**Table 2:** Comparison of meteorological data by DDT and AD type.

	**Wind (m/s)**				**Pressure (hPa)**			
	**DDT**	**AD**	**t/r**	***p***	**DDT**	**AD**	**t/r**	***p***
	**M ± SD**	**M ± SD**			**M ± SD**	**M ± SD**		
Total		14.6 ± 3.3	−2.279	**0.023**	835.5 ± 2.4	834.8 ± 2.2	3.835	**0.000**
			0.028	**0.000**			−0.023	
Period								
1st	15.4 ± 3.81	15.2 ± 3.38	2.155	**0.031**	836.3 ± 2.56	835.1 ± 2.53	2.509	**0.011**
2nd	13.4 ± 3.26	13.7 ± 3.09	−2.904	**0.004**	834.5 ± 1.91	834.6 ± 1.67	−1.628	0.104
Season								
Spring	16.3 ± 3.19	15.9 ± 2.58	2.955	**0.003**	833.6 ± 1.41	833.9 ± 1.38	−1.755	0.079
Summer	15.6 ± 3.85	15.5 ± 3.75	0.085	0.932	833.5 ± 1.37	833.6 ± 1.44	−1.725	0.085
Autumn	13 ± 2.46	13.1 ± 2.44	−1.077	0.282	837.5 ± 1.7	837.4 ± 1.56	1.175	0.240
Winter	12.8 ± 3.65	13.1 ± 3.24	−2.333	**0.020**	836.9 ± 1.85	836.5 ± 1.75	4.879	**0.000**
Month								
1	11.1 ± 1.03	11.2 ± 0.96	−0.099	0.921	836.3 ± 2.16	835.8 ± 1.95	3.313	**0.001**
2	14.3 ± 2.75	14.2 ± 2.64	0.607	0.544	832.4 ± 0.91	832.3 ± 0.8	2.333	**0.01**
3	17.1 ± 3.31	16.7 ± 2.55	2.529	**0.012**	834.1 ± 0.93	833.9 ± 0.83	3.943	**0.000**
4	15.4 ± 3.43	14.7 ± 2.94	2.991	**0.003**	834.5 ± 1.58	834.3 ± 1.46	0.850	0.423
5	16 ± 2.49	16.1 ± 1.99	−1.078	0.282	832.6 ± 0.82	833 ± 0.74	−0.920	0.379
6	18.2 ± 4.33	17.5 ± 4.14	2.274	**0.023**	833.7 ± 1.27	834.1 ± 1.29	−1.120	0.122
7	15.6 ± 2.67	15.8 ± 2.65	−1.059	0.29	836 ± 1.15	835.9 ± 1.06	0.75	0.454
8	13.3 ± 1.84	13 ± 1.9	1.896	0.058	834.6 ± 0.9	834.8 ± 0.68	−2.673	0.080
9	12.3 ± 1.7	12.7 ± 2.09	−2.224	**0.026**	835.8 ± 1.42	835.9 ± 1.36	−1.065	0.287
10	13.4 ± 2.98	13.3 ± 3.05	0.599	0.55	838.9 ± 1.03	838.7 ± 0.88	2.49	**0.014**
11	12.4 ± 2.25	13.1 ± 2.08	−4.099	**0.000**	837.7 ± 1.03	837.3 ± 1.02	5.191	**0.000**
12	12.9 ± 5.05	14.1 ± 4.49	−3.266	**0.001**	838.3 ± 1.08	838.2 ± 0.95	1.799	0.074
	**Moisture (%)**				**Temperature (°C)**			
	**DDT**	**AD**	**t/r**	***p***	**D**	**A**	**t/r**	***p***
	**M ± SD**	**M ± SD**			**M ± SD**	**M ± SD**		
Total		61.7 ± 14.1	−1.823	0.085	8.8 ± 10.3	8.7 ± 10.1	0.080	0.936
			0.006	0.352			−0.001	0.932
Period								
1st	66.5 ± 10.86	65.6 ± 10.89	2.727	**0.006**	4.9 ± 9.47	5.6 ± 9.5	−2.493	**0.001**
2nd	55.8 ± 16.28	56.3 ± 16.13	0.845	0.398	12.6 ± 9.65	13.2 ± 9.43	−1.578	0.115
Season								
Spring	67.4 ± 8.01	65.9 ± 8.32	4.275	**0.000**	7.3 ± 5.23	7.9 ± 5.44	−2.863	**0.004**
Summer	46.4 ± 9.95	46.5 ± 9.84	−0.147	0.883	21.4 ± 2.25	21.3 ± 2.15	0.518	0.604
Autumn	56.5 ± 14.35	58.5 ± 13.62	−2.787	**0.005**	11.1 ± 6.07	11 ± 5.61	0.301	0.764
Winter	73.9 ± 8.39	74.1 ± 7.18	−0.665	0.506	−4.2 ± 3.95	−4.1 ± 3.93	−0.431	0.666
Month								
1	72.2 ± 10.92	73 ± 8.72	−1.190	0.052	−5.8 ± 4.13	−5.3 ± 4.16	−1.542	0.123
2	73.7 ± 6.87	73.2 ± 6.41	1.330	0.120	−5 ± 3.03	−4.8 ± 3.3	−0.458	0.648
3	74.5 ± 5.08	73.9 ± 4.63	1.583	0.114	0.9 ± 2.39	1.5 ± 3.17	−3.041	**0.003**
4	61.5 ± 6.46	58.3 ± 5.99	6.678	**0.000**	9.2 ± 1.98	10.2 ± 1.92	−6.594	**0.000**
5	64.7 ± 5.59	64.4 ± 5.88	0.782	0.434	12.2 ± 1.41	12.4 ± 1.43	−1.986	**0.047**
6	51.8 ± 7.44	50.5 ± 7.1	1.445	0.145	18.9 ± 0.9	19.2 ± 0.75	−4.031	**0.000**
7	50.3 ± 7.16	50.8 ± 7.56	−0.923	0.256	21.7 ± 0.53	21.7 ± 0.59	−0.546	0.585
8	35.7 ± 6.29	35.3 ± 4.88	1.051	**0.210**	23.6 ± 2	24 ± 1.64	−1.43	0.216
9	40.5 ± 3.46	41.5 ± 3.6	−2.645	**0.009**	19.2 ± 0.7	19 ± 0.64	2.848	**0.005**
10	55.1 ± 11.99	59.2 ± 10.74	−4.069	**0.000**	11.4 ± 1.51	11 ± 1.2	3.859	**0.000**
11	70 ± 5.21	70.6 ± 4.48	−1.603	0.111	4.9 ± 1.06	5 ± 1.01	−1.908	0.101
12	76.6 ± 5.55	76.9 ± 4.67	−0.751	0.453	−1.9 ± 3.42	−1.5 ± 3.15	−1.519	0.131

DTT: Number of patients diagnosed with dermatitis, AD: Number of patients diagnosed with AD, 1st: January–June, 2nd: July–December, M: Mean, SD: Standard deviation, t: Independent samples *t*-test according to dermatitis type, Bold: *p* < 0.05.

**Table 3:** Comparison of air pollution data by DDT and AD type.

	**PM_10_**				**SO_2_**			
	**DDT**	**AD**	**t/r**	***p***	**DDT**	**AD**	**t/r**	***p***
	**M ± S**	**M ± S**			**M ± S**	**M ± S**		
Total		56.9 ± 35.3	4.564	**0.000**	13.8 ± 11.7	13.0 ± 11.4	2.862	**0.004**
			−0.019	**0.005**			−0.017	**0.011**
Period								
1st	59.2 ± 40.2	55.9 ± 34.9	2.806	**0.005**	15.5 ± 12	14.1 ± 11.5	3.849	**0.000**
2nd	62 ± 39.1	58.3 ± 35.6	2.693	**0.007**	12.1 ± 11.1	11.5 ± 11.2	1.466	0.143
Season								
Spring	40.5 ± 13.4	41.4 ± 11.7	−1.552	0.121	10.6 ± 7.2	8.9 ± 5.92	5.895	**0.000**
Summer	40.3 ± 10.4	41.4 ± 10.9	−1.450	0.118	3.2 ± 1.9	2.9 ± 1.24	3.294	**0.001**
Autumn	60.9 ± 25.7	55.1 ± 23.3	4.472	**0.000**	10.7 ± 6.5	10.4 ± 6.3	0.946	0.344
Winter	100.1 ± 52.9	91.1 ± 49.4	3.829	**0.000**	30 ± 6.67	29.6 ± 6.2	1.356	0.175
Month								
1	131.1 ± 44.8	116.6 ± 49.6	4.208	**0.000**	33.6 ± 5.5	32.3 ± 5.8	3.243	**0.001**
2	58.9 ± 15.3	55.6 ± 13.4	2.954	**0.003**	24 ± 4.95	24.7 ± 4.3	−1.921	0.055
3	46.7 ± 12.3	46.4 ± 8.8	0.39	0.697	18.5 ± 4.7	15.7 ± 4.1	8.061	**0.000**
4	40.6 ± 13.8	47.1 ± 12.1	−6.061	**0.000**	8.5 ± 2.99	8.1 ± 2.51	1.758	0.079
5	33.3 ± 10.1	32.9 ± 8.1	0.621	0.535	3.5 ± 0.37	3.4 ± 0.35	3.498	**0.000**
6	40.7 ± 13.3	43.6 ± 12.8	−2.99	**0.003**	2.4 ± 0.36	2.4 ± 0.29	−1.269	0.204
7	37.4 ± 5.8	38.4 ± 5.8	−2.467	**0.014**	2.7 ± 0.56	2.8 ± 0.54	−1.349	0.178
8	40.3 ± 10.9	42.4 ± 12.2	−2.101	**0.036**	4.7 ± 2.84	4 ± 2	3.121	**0.002**
9	44.1 ± 16	42.5 ± 15.6	1.059	0.29	3.9 ± 1.64	3.6 ± 1.4	1.726	0.085
10	62.6 ± 18.4	54.1 ± 16.7	5.489	**0.000**	7.7 ± 0.95	7.4 ± 0.7	3.333	**0.001**
11	72.5 ± 29.9	65.5 ± 28.4	2.744	**0.006**	18.5 ± 1.9	18.4 ± 1.5	0.549	0.583
12	108.8 ± 58	106.8 ± 52.3	0.403	0.687	32 ± 5.2	32.7 ± 4.5	−1.699	0.09
	**NO_2_**				**NO_x_**			
	**DDT**	**AD**	**t/r**	***p***	**DDT**	**AD**	**t/r**	***p***
	**M ± S**	**M ± S**			**M ± S**	**M ± S**		
Total		11.1 ± 4.7	3.760	**0.000**	17.2 ± 6.7	16.4 ± 6.6	4.707	**0.000**
			−0.024	**0.001**			−0.033	**0.000**
Period								
1st	11.4 ± 4.06	10.9 ± 3.95	4.264	**0.000**	16.1 ± 5.17	15.3 ± 4.95	4.988	**0.000**
2nd	11.7 ± 4.27	11.6 ± 4.43	0.484	0.629	18.2 ± 7.85	18 ± 8.18	0.798	0.425
Season								
Spring	9.8 ± 2.65	9.1 ± 2	6.131	**0.000**	14.1 ± 3.42	13.2 ± 2.55	6.270	**0.000**
Summer	7.1 ± 1.04	7 ± 1	2.218	**0.027**	9.8 ± 1.47	9.7 ± 1.39	2.050	**0.04**
Autumn	13.3 ± 2.42	13.4 ± 2.5	−0.999	0.318	20.9 ± 5.1	20.9 ± 5.25	0.019	0.985
Winter	16 ± 3.03	16 ± 2.82	−0.345	0.73	23.7 ± 4.5	23.3 ± 4.63	1.836	0.066
Month								
1	16.2 ± 3.8	16.3 ± 3.44	−0.472	0.637	23.6 ± 2.86	23.2 ± 2.79	1.878	0.061
2	15 ± 1.67	14.8 ± 1.6	1.441	0.15	19.6 ± 1.25	19.4 ± 1.16	1.611	0.107
3	12.4 ± 2.37	11.3 ± 1.65	6.712	**0.000**	17.4 ± 3.19	15.9 ± 2.23	6.546	**0.000**
4	8.8 ± 1.3	8.5 ± 1.24	2.899	0.004	13.1 ± 1.54	12.7 ± 1.45	3.109	**0.002**
5	7.7 ± 0.73	7.7 ± 0.74	0.099	0.921	11.2 ± 0.7	11.2 ± 0.73	−0.018	0.986
6	7.5 ± 0.96	7.2 ± 0.97	4.866	**0.000**	10.5 ± 1.32	10.1 ± 1.36	4.338	**0.000**
7	6 ± 0.3	6 ± 0.32	−0.797	0.425	8.2 ± 0.33	8.3 ± 0.33	−2.451	**0.014**
8	7.8 ± 0.54	7.9 ± 0.5	−1.059	0.29	11 ± 0.24	10.9 ± 0.2	1.964	0.05
9	10.2 ± 1.32	10 ± 1.34	1.828	0.068	14.2 ± 1.78	13.6 ± 1.84	2.987	**0.003**
10	13.3 ± 0.62	13.5 ± 0.53	−3.257	0.001	20.7 ± 1.2	20.6 ± 1.05	0.802	0.423
11	15.6 ± 1.12	15.8 ± 0.96	−2.87	0.004	26.2 ± 1.18	26.5 ± 0.94	−3.294	**0.001**
12	16.6 ± 2.96	17.2 ± 2.64	−2.315	0.021	27.5 ± 4.32	28.7 ± 4.07	−3.303	**0.001**
	**NO**				**O_3_**			
	**DDT**	**AD**	**t/r**	***p***	**DDT**	**AA**	**t/r**	***p***
	**M ± S**	**M ± S**			**M ± S**	**M ± S**		
Total		5.2 ± 2.9	5.301	**0.000**	70.4 ± 29.9	74.9 ± 29.7	−6.600	**0.000**
			−0.030	**0.000**			0.051	**0.000**
Period								
1st	4.7 ± 1.76	4.4 ± 1.47	4.868	**0.000**	72.8 ± 19.3	77.1 ± 19.8	−7.459	**0.000**
2nd	6.5 ± 3.94	6.4 ± 4.03	1.074	0.283	68.1 ± 37.4	71.9 ± 39.5	−2.87	**0.004**
Season								
Spring	4.3 ± 0.81	4.1 ± 0.6	6.434	**0.000**	77.3 ± 14	83 ± 13.8	−9.800	**0.000**
Summer	2.7 ± 0.66	2.7 ± 0.58	1.038	0.299	107.6 ± 22.9	110.5 ± 22.1	−2.726	**0.006**
Autumn	7.6 ± 3.05	7.5 ± 3.04	0.83	0.407	47.5 ± 14.3	47.9 ± 13.5	−0.552	0.581
Winter	7.8 ± 3.39	7.3 ± 3.33	2.75	**0.006**	49 ± 14.8	51.3 ± 13.8	−3.421	**0.001**
Month								
1	7.4 ± 2.21	6.9 ± 1.77	3.297	**0.001**	48.9 ± 12.7	51.8 ± 10.7	−3.09	**0.002**
2	4.6 ± 0.55	4.6 ± 0.57	−0.748	0.455	62.4 ± 11.3	61.1 ± 11.6	1.476	0.14
3	5 ± 0.82	4.6 ± 0.59	6.107	**0.000**	69.1 ± 10.6	75.7 ± 8.4	−8.862	**0.000**
4	4.3 ± 0.26	4.2 ± 0.23	3.961	**0.000**	76.4 ± 5.3	78.3 ± 6.2	−4.471	**0.000**
5	3.5 ± 0.09	3.5 ± 0.07	−1.494	0.135	87.7 ± 16.3	92.9 ± 15.7	−4.715	**0.000**
6	3 ± 0.43	2.9 ± 0.44	2.39	**0.017**	94.7 ± 11.1	98.5 ± 9.8	−4.789	**0.000**
7	2.2 ± 0.38	2.2 ± 0.36	−1.452	0.147	125.1 ± 24.3	129.2 ± 23.2	−2.201	**0.028**
8	3.1 ± 0.66	3 ± 0.55	1.566	0.118	99.8 ± 16	102.1 ± 15.5	−1.661	0.097
9	3.9 ± 1.23	3.6 ± 1.13	2.325	**0.02**	67.3 ± 5.1	67.8 ± 5	−1.129	0.259
10	7.4 ± 1.68	7.2 ± 1.5	1.784	0.075	45.2 ± 1.7	45 ± 1.6	1.305	0.192
11	10.6 ± 0.8	10.7 ± 0.7	−0.626	0.532	34.1 ± 5.1	35.8 ± 5.1	−3.849	**0.000**
12	10.9 ± 2.9	11.6 ± 2.7	−2.51	**0.012**	37 ± 7	37.3 ± 5.8	−0.544	0.586

D: Number of patients diagnosed with dermatitis, A: Number of patients diagnosed with AD, 1st: January–June, 2nd: July–December, M: Mean, SD: Standard deviation, t: Independent samples *t*-test according to dermatitis type, Bold: *p* < 0.05. Note: Air pollution data represent monthly mean values.

**Table 4:** Binary Logistic Regression Analysis Results for AD.

	B	S.E.	Wald	df	Sig.	Exp(B)	95% C.I. for EXP(B)	
							Lower	Upper
Demographics								
Gender (1:Male)	−0.249	0.047	28.557	1	0.000 *	0.779	0.711	0.854
Age	−0.104	0.005	366.494	1	0.000 *	0.901	0.891	0.911
Period								
Period (1: First half of the year)	−0.466	0.076	38.003	1	0.000 *	0.628	0.541	0.728
Meteorological								
Pressure	0.11	0.022	24.266	1	0.000 *	1.117	1.069	1.167
Wind Value	0.006	0.009	0.475	1	0.491	1.006	0.989	1.023
Air pollution								
PM_10_	−0.004	0.001	14.578	1	0.000 *	0.996	0.994	0.998
O_3_	0.015	0.002	65.06	1	0.000 *	1.015	1.011	1.018
SO_2_	−0.007	0.007	0.907	1	0.341	0.993	0.98	1.007
NO_2_	0.436	0.716	0.372	1	0.542	1.547	0.381	6.289
NO_X_	−0.381	0.714	0.284	1	0.594	0.683	0.169	2.771
NO	0.4	0.714	0.314	1	0.575	1.491	0.368	6.041
(Constant)	−95.228	18.932	25.3	1	0.000			
			**DDT**		**AD**		**Percentage Correct**	
Categories	DDT		19,256		0		100.0	
	AD		2151		0		0.0	
			Overall Percentage				90.0	

Model Omnibus Chi-square = 660.230 (df = 11, *p* < 0.0001); –2 Log Likelihood = 13,304.724; Cox and Snell R^2^ = 0.030; Nagelkerke R^2^ = 0.063; Hosmer–Lemeshow Chi-square = 44.650 (*p* < 0.0001). * *p* < 0.001.

**Table 5:** Model Summary for Regression Trees.

Specifications	Growing Method	CHAID
	Dependent Variable	Categories (DDT vs. AD)
	Independent Variables	Gender, Period, Age, Pressure Value, PM_10_, O_3_
	Validation	Cross Validation Resubstitution Estimate = 0.100 ± 0.002 Cross-Validation Estimate = 0.100 ± 0.002 Risk estimate = 10.0%
	Maximum Tree Depth	3
	Minimum Cases in Parent Node	100
	Minimum Cases in Child Node	50
Results	Independent Variables Included	Age, Period, O_3_
	Number of Nodes	13
	Number of Terminal Nodes	7
	Depth	3
Classification matrix	DDT	100.0% accuracy
	AD	0.0% accuracy
	Overall Percentage	90.0%

## Data Availability

The data used in this study are available from the corresponding author upon reasonable request.

## Abbreviations

AD	Atopic Dermatitis
CHAID	The decision tree model
DDT	Different Dermatitis Types
PM_10_	Particulate matter with a diameter of 10 μm or smaller suspended in the air
O_3_	Ozone
NO_2_	Nitrogen Dioxide
SO_2_	Sulfur Dioxide
NOx	Nitrogen Oxides
